# Phonetic entrainment in L2 human-robot interaction: an investigation of children with and without autism spectrum disorder

**DOI:** 10.3389/fpsyg.2023.1128976

**Published:** 2023-06-19

**Authors:** Yitian Hong, Si Chen, Fang Zhou, Angel Chan, Tempo Tang

**Affiliations:** ^1^Department of Chinese and Bilingual Studies, The Hong Kong Polytechnic University, Hong Kong, Hong Kong SAR, China; ^2^Department of Chinese and Bilingual Studies, Research Centre for Language, Cognition, and Neuroscience, The Hong Kong Polytechnic University, Hong Kong, Hong Kong SAR, China; ^3^Research Institute for Smart Ageing, The Hong Kong Polytechnic University, Hong Kong, Hong Kong SAR, China; ^4^The HK PolyU-PekingU Research Centre on Chinese Linguistics, The Hong Kong Polytechnic University, Hong Kong, Hong Kong SAR, China

**Keywords:** phonetic entrainment, autism, children, robot, controlled speech, conversation task

## Abstract

Phonetic entrainment is a phenomenon in which people adjust their phonetic features to approach those of their conversation partner. Individuals with Autism Spectrum Disorder (ASD) have been reported to show some deficits in entrainment during their interactions with human interlocutors, though deficits in terms of significant differences from typically developing (TD) controls were not always registered. One reason related to the inconsistencies of whether deficits are detected or not in autistic individuals is that the conversation partner’s speech could hardly be controlled, and both the participants and the partners might be adjusting their phonetic features. The variabilities in the speech of conversation partners and various social traits exhibited might make the phonetic entrainment (if any) of the participants less detectable. In this study, we attempted to reduce the variability of the interlocutors by employing a social robot and having it do a goal-directed conversation task with children with and without ASD. Fourteen autistic children and 12 TD children participated the current study in their second language English. Results showed that autistic children showed comparable vowel formants and mean fundamental frequency (*f0*) entrainment as their TD peers, but they did not entrain their *f0* range as the TD group did. These findings suggest that autistic children were capable of exhibiting phonetic entrainment behaviors similar to TD children in vowel formants and *f0*, particularly in a less complex situation where the speech features and social traits of the interlocutor were controlled. Furthermore, the utilization of a social robot may have increased the interest of these children in phonetic entrainment. On the other hand, entrainment of *f0* range was more challenging for these autistic children even in a more controlled situation. This study demonstrates the viability and potential of using human-robot interactions as a novel method to evaluate abilities and deficits in phonetic entrainment in autistic children.

## 1. Introduction

During conversations, the interlocutors from a typical population coordinate with each other in verbal and non-verbal ways. These cooperative behaviors—where individuals adjust their behaviors to match closely mirror their conversation partners—are referred to as entrainment (also called “convergence,” “alignment,” or “accommodation” in some studies). For example, it has been found that interlocutors who are strangers to one another use head nodding and eye gaze coordination to signal mutual understanding (Cassell et al., 2007). On the other hand, entrainment in speech is more subtle and complicated. Studies working on phonetic entrainment have adapted diverse cooperative tasks and involved a wide variety of speech features. For example, a series of English and Mandarin corpus studies revealed similar *f0*, intensity, and speech rate between interlocutors when they were playing computer games that required communication (Levitan and Hirschberg, 2011; Xia et al., 2014; Levitan et al., 2015). In addition, children as young as 9 years old were found to converge in mean *f0* in “spot-the-difference” games in Lehnert-LeHouillier et al. (2020). Hogstrom et al. (2018) also reported convergence of phoneme duration from children aged from 12 to 18 in a cooperative map searching task.

In essence, phonetic entrainment is the product of the connection between perception and production (Coles-Harris, 2017). The process of phonetic entrainment requires the ability to detect the acoustic cues of the interlocutor(s) and adjust one’s own production accordingly (Phillips-Silver et al., 2010; Wynn et al., 2018). From this perspective, deficits in speech prosody might cause failure in phonetic entrainment. Atypical prosodic production—such as wider *f0* range (Nadig and Shaw, 2012) and longer turn-taking gaps (Ochi et al., 2019)—was found in individuals with Autism Spectrum Disorder (ASD). Studies on phonetic entrainment of autistic children showed mixed results. Hogstrom et al. (2018) found that TD children converged in their phoneme duration in the post-interaction period while autistic children showed a trend of divergence. However, some studies reported a tendency of similar unchanged phonetic adjustment between autistic and TD children, for example, similar unchanged adjustment in speech rate (Wynn et al., 2018) and *f0* range (Lehnert-LeHouillier et al., 2020).

One reason for the undetected phonetic entrainment in children might be due to the fact that the required prosodic skills have not developed into an adult-like level (Wynn et al., 2018). Another reason is that both participants and conversation partners have the potential to adjust their phonetic features at the same time, which makes it harder to examine phonetic convergence from one side. Additionally, variation in conversation partners, such as their various social traits, might make the phonetic entrainment (if any) of the participants more varied and with less detectable patterns. Furthermore, as previous studies have indicated, the age range of 7–12 is a critical period for children’s development of rhythm recognition (Upitis, 1987). Therefore, if the speech of their interlocutors can be controlled with no phonetic adjustment and no variations throughout the experiments, we might be able to detect phonetic entrainment patterns in autistic children. This possibility has not been available until the application of social robots.

In this study, we use a social robot as a conversation partner to investigate whether phonetic entrainment can be found among children with and without ASD in a conversation task with a better controlled interlocutor. During the experiment, the acoustic features and social traits (reflected in facial expressions and the manner of interactions) of the robot remained consistent. Children’s conversations with the robot were recorded and compared with their baseline production and post-interaction production. We target at the entrainment of fundamental frequency (*f0*), and formant frequencies (F1, F2). *F0* refers to the vibration of vocal folds (Yavas, 2011). The perceptual correlate of *f0* is pitch, which reveals signals of sound identity and information about meaning (McPherson and McDermott, 2018). The variation of *f0* is an important part of speech prosody manipulation. By examining mean *f0* and *f0* range, we can understand more about their adjustment of pitch during interaction. Formant frequencies relates to vocal tract configuration, reflecting the tongue position when the speaker articulates the vowels (Yavas, 2011). The investigation of first and second vowel formant improves our understanding about vowel space area manipulation during conversations (Pettinato et al., 2016).

## 2. Background

### 2.1. Entrainment in the broad sense

Humans show an in-born tendency to coordinate with outside stimuli (Phillips-Silver et al., 2010). For example, humans tend to clap hands or shake heads along with the rhythm of a song when they are exposed to it. Infants as young as 5 months old have shown coordinated body movement with music (Ilari, 2015). Such coordination is called entrainment.

Social entrainment occurs when the outside stimulus comes from another human (Phillips-Silver et al., 2010). During the social interaction, social entrainment demonstrates social functions important in facilitating social communication. By entraining in the time domain (e.g., entrainment of turn-taking gaps), it improves mutual understanding between the interlocutors, helps build consensus and establish positive connections (Borrie and Liss, 2014). It fulfills the function of sustaining the emotional and social relationship between interlocutors (Borrie and Liss, 2014). Social entrainment also increases the interlocutors’ enjoyment of the communication and facilitates the development of the social relationship. In the study of Chartrand and Bargh (1999), they asked the interlocutor to intentionally mimic the gestures of the participants and asked the participants to rate the experience of social interaction after the experiment. They found that when interlocutors mimicked participants’ gestures, the participants rated the experience as smoother and the interlocutor as friendlier compared to the control group (where the interlocutors did not mimic any gestures). In regard to phonetic entrainment, Borrie and Delfino (2017) found that interlocutor dyads who showed a match of vocal fry frequency tended to find their communication more enjoyable. In a corpus study, Lee et al. (2010) demonstrated that couples with positive emotions during conversations showed *f0* related entrainment as compared to those with negative emotions. Furthermore, social entrainment increases communication efficiency. It improves information transfer and enhances mutual agreement and sympathy (Gill, 2012). In the same study, Borrie and Delfino (2017) found that participants’ degree of entrainment on their frequency of vocal fry was also positively correlated with their efficiency in doing a cooperative task. Similarly, Nenkova et al. (2008) found that entrainment of high frequency lexicons led to higher scores in cooperative games. More specifically, Levitan et al. (2011) found that entraining backchannel cues decreased turn-taking gaps and interruptions and improved task complication efficiency.

Since phonetic entrainment might be correlated with social rapport and social communication efficiency, deficits in entrainment could be associated with poor social skills. Therefore, populations with communication disorders are more likely to have deficits in entrainment. Autism Spectrum Disorder (ASD) is one group of disorder correlated with communication and social interaction difficulties. According to American Psychiatric Association (2022), individuals with ASD demonstrate three core characteristics: atypical social communication, restricted interests, and repetitive behaviors. Empirical evidence has shown that autistic populations did present certain degrees of deficits in social entrainment. Previous studies have found that autistic individuals did not show comparable non-verbal social entrainment relative to their TD peers. Nakano et al. (2011) found that autistic adults failed to entrain their eyeblink with the speakers. The eyeblink entrainment occurred at conversation pause, forming an important part of conversation coordination. The disruption of eyeblink entrainment might affect autistic individuals’ social interactions. Other than the eyeblink, autistic individuals also demonstrated incomparable facial muscle movement when mimicking others’ emotions, which was suggested to affect their social reciprocity with conversation partners (Mathersul et al., 2013). Similarly, Yoshimura et al. (2015) reported reduced times of facial expression synchrony of autistic individuals as compared to the TD population. They found that individuals with higher degree of social dysfunction tended to show lower frequency of facial expression synchrony. In a different study working with autistic children, Helt et al. (2010) reported delays in development in yawning mimicry. They suggested that autistic children’s delayed acquisition of social behavior mimicry might be due to the lack of social interest in interaction, and in turn, they have fewer social experiences compared to their TD peers.

These behaviors are categorized as contextual and socially meaningful entraining behavior, distinguishable from simple automatic mimicry (Nakano et al., 2011). The breakdown of such behaviors could potentially be associated with unpleasant and ineffective social communication. On the other hand, the model of social entrainment might provide a new perspective for understanding autistic populations’ social behaviors.

### 2.2. Speech features and phonetic entrainment of autistic children

Unlike non-verbal entrainment, entrainment in phonetic features is a more fine-grained process, where the interlocutors detect and perceive the phonetic features (e.g., speech rate, fundamental frequency (*f0*), vowel formant) of their conversation partners and adjust their own phonetic features in speech production accordingly. This process involves the processes of phonetic perception and production. Atypicality in any step of this process might lead to deficits in phonetic entrainment. The autistic population has long been found to show different speech features from the TD population, such as vowel formants and *f0* range. Although the reasons behind their atypical speech features remain unclear, the empirical studies working on gaining a better understanding of their speech features might provide some hints on their phonetic entrainment.

Studies on vowels mainly reported exaggerated vowel formants produced by autistic children. Lyakso et al. (2016) found larger vowel formant triangles in autistic children when compared to their TD peers. Mohanta and Mittal (2022) reported higher vowel formants for autistic children than TD children, which was interpreted as atypicality in vowel production mechanism. However, their production tended to show less dispersion. Kissine and Geelhand (2019) and Kissine et al. (2021) reported lower variabilities of vowel formants in autistic children, compared to their *f0*. They proposed a possibility that autistic individuals tended to pay more attention to the precision of vowel pronunciation and thus might overact the target articulatory manners, leading to exaggerated vowel formants, while TD individuals spoke in a more leisure style.

In regard to speech prosody, discrepancies exist between the findings from production and perception. Nadig and Shaw (2012) found a significantly larger *f0* range in the autistic group than TD group, but no significant difference in the mean *f0*. The larger pitch range of the autistic population, although acoustically abnormal, was not perceived as a signal of odd speech by TD listeners (Nadig and Shaw, 2012). Similarly, Patel et al. (2020) found larger *f0* excursion in utterance-final position, but it did not serve as a marker of autism to non-clinical listeners. However, in contrast to this, Shriberg et al. (2001) reported that over half of the autistic participants were rated as exhibiting unusual prosody while only about 6% in TD participants were rated the same. This finding was associated with differences in the mean *f0* and *f0* range between these two groups. The mixed findings of perceptual differences of their prosody indicated that it was difficult for listeners to interpret the prosodic cues of autistic individuals. This might be due to the fact that autistic population did not use prosody functionally in communication (Nadig and Shaw, 2012). They tended to use a limited repertoire of prosody repetitively, which may be related to one of their core features—restricted and repetitive behaviors (Green and Tobin, 2009). On the other hand, the exaggerated style of prosody (higher *f0* and larger *f0* range) is similar to infant-directed speech, which is suggested to be a signal of inability to outgrow from motherese, indicating their undeveloped control of prosody (Sharda et al., 2010). These two indications point to a possibility that autistic individuals are less flexible in adjusting prosodic features in communication relative to their TD peers. Therefore, it is suspected that their entrainment in prosodic cues might not be as comparable as their TD peers.

Some studies have revealed lack of entrainment in a variety of phonetic features from autistic adults in their first language. For instance, no flexible adjustment of speech volume by autistic adults was reported in Ochi et al. (2019). Autistic adults were also found to lack speech rate entrainment in a quasi-conversation experiment as opposed to their TD peers (Wynn et al., 2018). However, in terms of studies of entrainment from autistic children, the results were inconsistent. In the study of Wynn et al. (2018), although they found significant differences of speech rate convergence between autistic and TD adults, autistic children and TD children’s speech rate did not show significant differences. Hogstrom et al. (2018) compared the phoneme duration of keywords before and after a conversation task with a TD interlocutor. They reported that autistic children tended to diverge in phoneme duration from the interlocutor after the task, compared with the pre-task production, while TD children showed convergence. However, they also found that neither autistic children dyads nor TD children dyads demonstrated *f0* adjustment. Lehnert-LeHouillier et al. (2020) reported no significant difference of *f0* range entrainment between autistic and TD teens. These findings suggest that we need to understand more about the conditions under which autistic children show or do not show TD-like phonetic entrainment, before one can better evaluate whether phonetic entrainment can serve as a linguistic biomarker for differentiating and TD children. Moreover, specifying these conditions inform us about their capabilities in achieving phonetic entrainment and their deficits in this aspect.

### 2.3. Phonetic entrainment in second language (L2)

Previous studies carrying out conversation task between L1 and L2 speakers reported more phonetic convergence from L2 speakers than their L1 interlocutors (Hwang et al., 2015). Similarly, in word shadowing task, L2 speakers are found with more phonetic convergence than L1 speakers (Lewandowski and Nygaard, 2018; Gnevsheva et al., 2021). They argued that larger phonetic differences between L1 and L2 speech allow L2 speakers to have more space for entrainment (Lewandowski and Nygaard, 2018). It can also be explained by a mediated priming effect with the intention of producing more native-like speech (Hwang et al., 2015), namely the more prestigious variety (Gnevsheva et al., 2021) and increasing communication efficiency. It remains unknown whether non-native autistic speakers demonstrated a similar pattern of L2 phonetic entrainment.

Although previous studies have reported a delay of autistic individuals’ L1 development, particularly in discourse and pragmatic functions (Kelley et al., 2006), there are studies reporting that their L2 was relatively unaffected (Práinsson, 2012; Agostini and Best, 2015). These studies mainly involved autistic subjects who did not suffer from intellectual impairment and whose language abilities were comparable with their TD peers in general. For example, the case study in Práinsson (2012) reported that the autistic subjects showed a good command of pragmatics, discourse prosody, and syntax of second language and even surpassed their TD peers. Agostini and Best (2015) found that the second language grammatical development of young autistic children was comparable and even faster than their TD peers. Because studies focusing on autistic individuals’ second language are scarce, and no study examined phonetic entrainment of their L2, this study attempts to provide some innovative empirical evidence of autistic children’s phonetic entrainment in their L2 to further our understanding of their second language acquisition.

### 2.4. Benefits of using a social robot as a conversation partner

As compared to entrainment in non-social contexts, such as entrainment with musical rhythm, social entrainment is special because it is a mutual process where both individuals adjust their behaviors to approximate each other’s. This special condition brings uncertainty and might be the reason why previous findings on the phonetic entrainment of autistic population tended to be inconsistent. Lehnert-LeHouillier et al. (2020) found that the atypical entrainment behavior of autistic youth, evidenced by a manipulation of difference between conversation dyads, was in fact the result of adjustment from their conversation partner. Therefore, the current study uses a social robot as a conversation partner to investigate the phonetic entrainment of autistic children in comparison with their TD peers. A social robot has the advantages of controlled speech with no phonetic entrainment and consistent social complexities, which might facilitate the detection of children’s phonetic adjustment.

Social robots have been used previously in therapy and research on autism in a longitudinal study, Robins et al. (2005) found that autistic children’s social skills were improved with the help of a humanoid robot. Dautenhahn and Werry (2004) found that autistic children showed more engagement in activities with robots and learned how to take turns and imitate the robot. Similar findings were reported by Barakova et al. (2015) where a robot-present scenario led to more social initiations of autistic children. Stanton et al. (2008) also found that autistic children were able to treat social robots as a social category and produce more words than playing with a non-verbal robot. In addition, some studies working on robotic voice have reported that autistic children exhibit a special preference to mechanic voices rather than human voices (Kuhl et al., 2005).

These attempts of using social robots to assist autistic populations reveal benefits, such as reducing the social pressure of autistic individuals and attracting their attention. Compared to human beings, social robots have fewer social complexities, e.g., more controlled facial expressions. They are more predictable due to their consistent voice and gestures (Marchi et al., 2014). They provide a structured interaction environment for autistic individuals to converse and learn (Kumazaki et al., 2020). These advantages of social robot might resolve the uncertainty of phonetic entrainment in human-human interactions. By designing an experiment of human-robot interaction, we aim to examine autistic children’s phonetic entrainment in a more controlled context.

### 2.5. Research questions and predictions

As reviewed above, autistic individuals might have problems in manipulating phonetic features in conversations. The inconsistency of interlocutors increases their difficulties in phonetic entrainment and also makes the phonetic manipulation of autistic individuals less detectable. The controlled nature of a robot provides a controlled conversation environment which might facilitate phonetic entrainment and its detection of autistic individuals. Moreover, as convergence on more speech features toward words recorded naturally than words generalized in synthetic voice has been reported (Gessinger et al., 2021), more natural speech used in the current study might trigger more entrainment than previous child-robot interaction studies (see Section 3.2.2. for more details about the sound used in the robot). Therefore, our main research question is: do autistic children and TD children show comparable phonetic entrainment when interacting with a social robot?

We expect that autistic children may show phonetic entrainment in a more controlled phonetic and social environment, but their performance may still be different from TD children. Specifically, we will examine vowel formants and fundamental frequency in the speech production of a group of autistic children and compare their production with their TD peers to identify whether they would show TD-like phonetic entrainment. We predict that autistic children are more likely to entrain vowel formant toward the standardized vowel target, consistently produced by the robot. On the other hand, we predict that their deficits of prosody will still affect their entrainment even when they interact with a controlled interlocutor. Therefore, they are predicted to show problems in phonetic entrainment of *f0*-related parameters (mean *f0* and *f0* range in the study).

## 3. Methods

Because phonetic entrainment is supposed to occur in both segmental level and prosodic level, the main task should be able to elicit natural conversational speech, and also yield enough repetitions for word-level acoustic analysis. We did not consider Map Tasks (Anderson et al., 1991), where one interlocutor found a route in the picture following the instruction of the other. Because the conversation dyads do not receive equal amount of information in the task, they have different pre-defined roles (i.e., a giver and a follower) and very uneven amount of production, which is not suitable for investigating phonetic entrainment. Therefore, we finally decided on a “spot the difference” game (van Engen et al., 2010). The main task was between the child and the robot, during which the participant interacted with a robot to find the differences between four pairs of pictures. The robot and the child participant would refer to pictures with slight differences in these four pairs. The robot asked questions regarding the color, number, and behavior of the objects in the pictures, guiding the child to notice the differences and to elicit keywords from him or her (see Section 3.2 for more details).

### 3.1. Participants

Fourteen L2 English-speaking autistic children and 12 age-matched typically developing (TD) children were recruited in Hong Kong. The autistic children received a clinical diagnosis of ASD from clinical settings in Hong Kong according to information provided by their parents. Both autistic and TD children had nonverbal IQ above 80 as assessed by the Raven’s Standard Progressive Matrices Test (Raven, 2003). These children acquire Cantonese as their first and home language, and English and Mandarin as their second languages at school. Since this study focused on L2 English, their spoken English was assessed by Comprehensive Assessment of Spoken Language (CASL; Carrow-Woolfolk, 2017) and autistic children showed moderate English language proficiency. There were no reported hearing impairments nor neurological disorders for all participants. As previous study has found that musical experience might affect perception of phonetic details (Tsang et al., 2018), the musical training experience of two groups was controlled to be comparable. Their chronological age, duration of musical training, IQ standard score, CASL standard score, age of English acquisition, and their English proficiency (out of 5 as the maximum score) reported by their parents are shown in Table 1. One TD child (t10) did not take the Raven Test or the CASL test, and she showed no sign of abnormality according to the observation of the experimenter. Parents of the participants signed a written consent form, which was approved by the Departmental Research Committee of the Hong Kong Polytechnic University, and the participants were reimbursed for participation.

**Table 1:** Means (and standard deviations) of chronological age, IQ standard score, CASL standard score, duration of musical training (months), age of English acquisition, and English proficiency score (5 points each) across the two groups of children.

Group	Number (male)	Chronological age	IQ score	CASL score	AoA	Musical training	Listening	Writing	Reading	Speaking
ASD	14 (9)	9.5 (1.16)	102.29 (14.79)	66.43 (21.25)	2.8 (1.65)	18 (21.05)	3.5 (0.76)	2.9 (1.07)	3.4 (1.01)	2.9 (1.07)
TD	12 (8)	9.1 (1.16)	103.2 (14.03)	88.8 (22.76)	2.6 (1.44)	22 (19.54)	4.1 (0.51)	3.5 (0.90)	3.8 (0.87)	3.5 (1.09)

### 3.2. Materials and procedures

#### 3.2.1. Pictures and keywords

**Figure 1:**
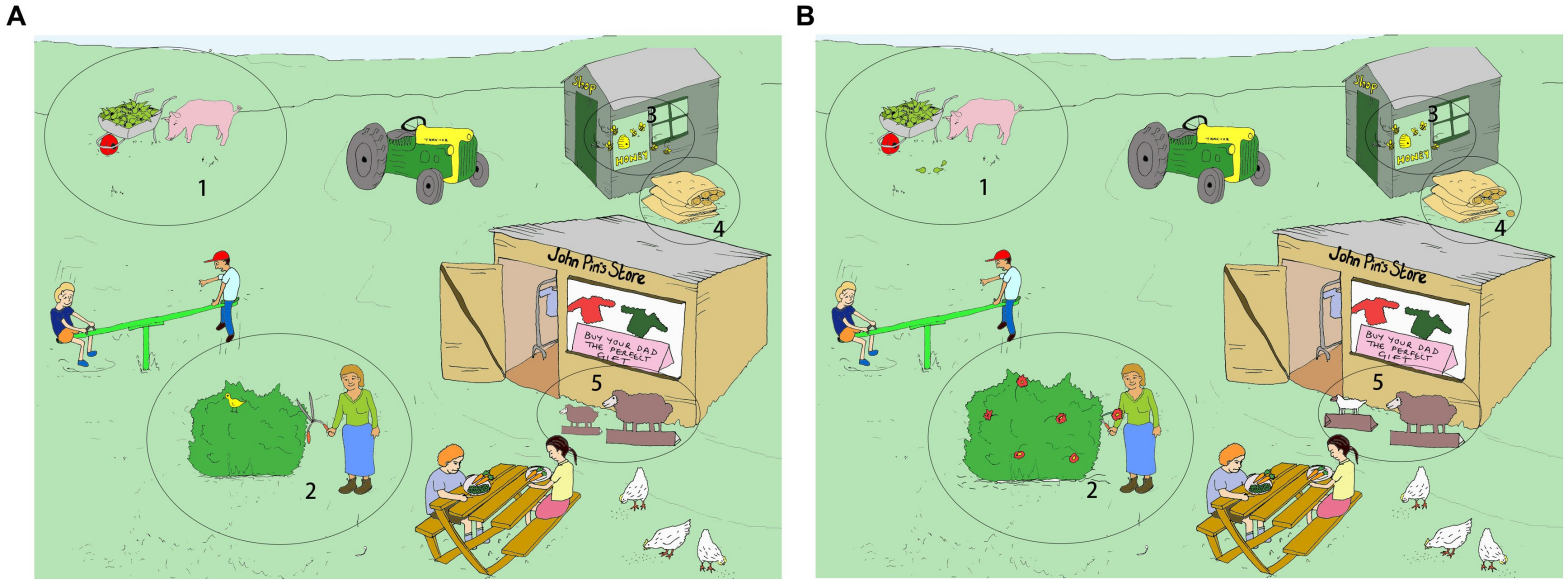
One of the four picture pairs for experiment. The child held picture **(A)**. The robot held picture **(B)**. Image source: https://doi.org/10.5281/zenodo.3703202. Reproduced under the terms of Creative Commons Attribution 4.0.

The task materials were adapted from pictures designed in DiapixUK tasks (Baker and Hazan, 2011). They are 12 pairs of cartoon pictures specially designed for “spot-the-difference” game in English. The pictures included three themes. Each theme has four pairs of pictures, sharing similar vocabulary and depicting the same keywords. The picture set depicting the farm theme was selected. There were originally 12 differences (depicting 12 different keywords) per pair in their design. This design has been used in studies with native speakers as young as 8 years old (Pettinato et al., 2016; Tuomainen et al., 2022). Given the condition of our participants (children with Autism Spectrum Disorder often have issues with executive function), we revised the pictures to reduce the number of differences to five, to reduce the level of task complexity. The differences related to either a change of the item (e.g., an apple and a pear in picture A vs. two pears in picture B; an empty sack in picture A vs. a full sack in picture B; white sheep in picture A vs. gray sheep in picture B) or an item that was missing in one picture (e.g., a bush with flowers in picture A vs. a bush without flowers in picture B). To increase visual saliency, the areas associated with the differences between a pair of pictures were circled and numbered in the pictures (see Figure 1 for a sample picture pair). The keywords related to the differences will be used for analyzing phonetic entrainment in segmental level while the conversational speech produced during interaction will be used for investigating prosodic entrainment.

#### 3.2.2. Robot and experimental setup

The robot we used in this study as a conversation partner is social robot Furhat (Al Moubayed et al., 2012). Furhat robot has a physical body with a neck and a movable head with a light-projected face. Its speech production was pre-scripted to be triggered by corresponding keywords. The robot’s speech was generated using Amazon Polly neural TTS system. Compared to usual robotic speech, their speech showed more naturalness in dialog due to shorter response time and higher articulation accuracy (Amazon Polly Developer Guide, 2023). In particular, we selected the voice of an American English male named Matthew which was produced by the neural TTS system rather than standard system. The neural system used a sequence-to-sequence method to generate “the most natural and human-like” sounds with rather higher quality (Amazon Polly Developer Guide, 2023, p. 1). The volume of the speech was set consistently for all the children.

**Figure 2:**
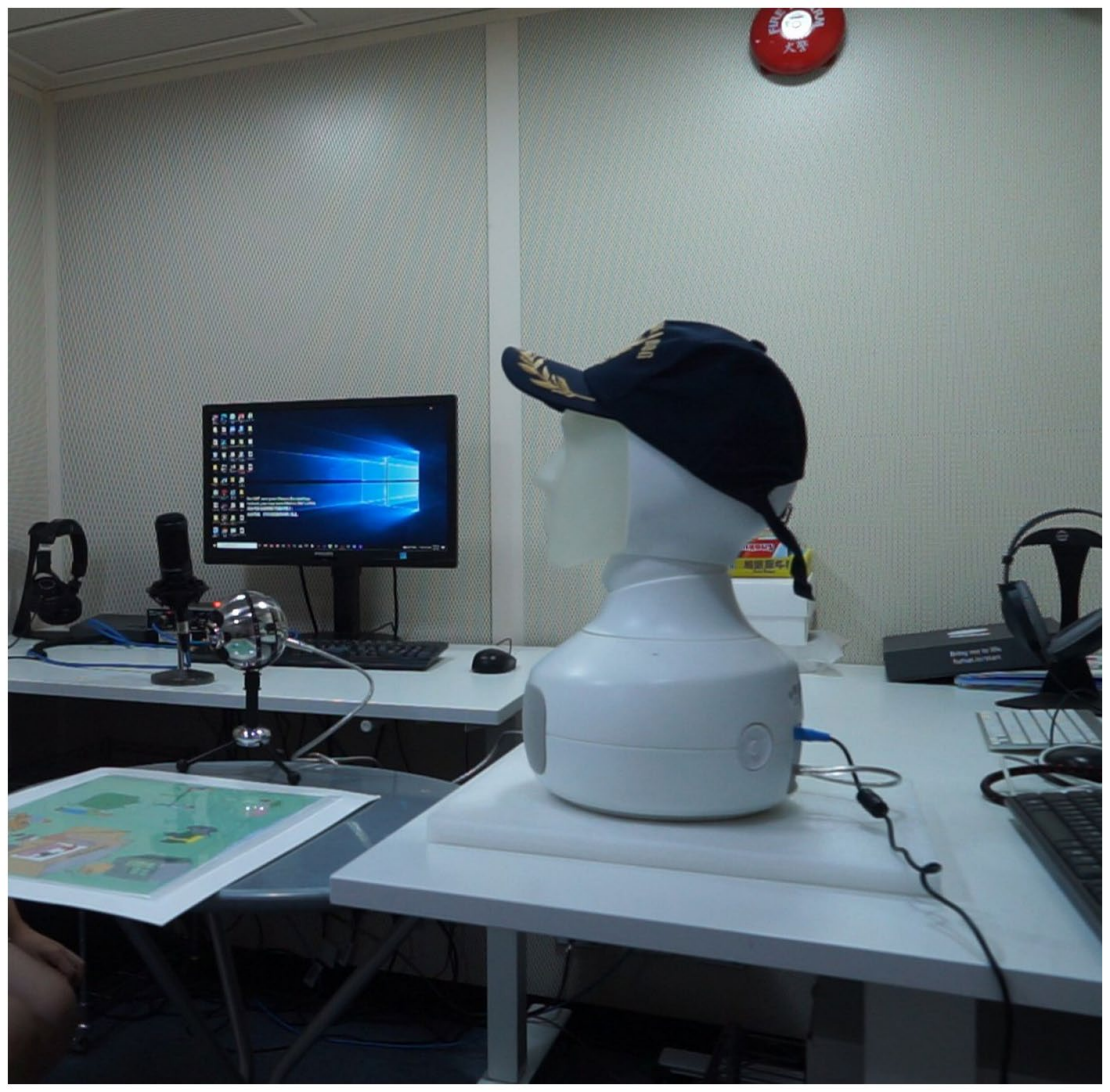
Experimental setting. Image source: https://doi.org/10.5281/zenodo.3703202. Reproduced under the terms of Creative Commons Attribution 4.0.

The experiment took place in a soundproof booth, and the robot was placed on a table about 85 cm away from the participant. The child participant sat facing the robot, and the seat height was adjusted to make sure that each child was at the robot’s eye level. The picture to elicit speech interaction and a microphone Blue Snowball connected to the robot were placed on a table in between the participant and the robot. The robot used the microphone to receive speech from the participant so as to trigger its corresponding response upon perceiving certain keywords. The speech recordings were done at a 44.1 kHz sampling rate with 16-bit resolution by another microphone, an Azden ECZ-990 microphone, connected with audacity in the computer. This recording microphone was placed on another table by the left of the participant. The experimental set-up is demonstrated in Figure 2.

### 3.3. Procedures

Before interacting with the robot, the child recorded five keywords as baseline production. They were shown pictures of keywords one by one on the screen and asked to say what they could see in the picture in English. Each keyword was produced in singular and plural forms, twice in isolation and once in a carrier sentence: I can see the KEYWORD(s) in the picture. Each keyword was elicited in 2 forms * (2 isolation + 1 carrier) = 6 repetitions. After baseline production, the child watched a video introducing how to play the ‘spot the difference’ game presented in their first language Cantonese to ensure they understood the task expectation well. Then, one pair of pictures depicting themes different from the experimental test items was given to the child for practice. To allow adequate time to let the child become familiarized with the task procedures, each child was given 5 min to try to determine the differences between the two pictures by themselves.

The interaction with the social robot started with a “say-hello” session. The robot greeted the child to familiarize the child with the robot’s voice. The “say-hello” session triggered four turns of interactions between the child and the robot. The experimenter double checked with the child to confirm their readiness before starting the interaction tasks. There are four pairs of pictures to look for differences (four tasks). These tasks were launched by the experimenter one at a time. The task order was randomized. Each task lasted for 10–15 min. The child was allowed to take a break between the tasks.

After the child finished all the tasks, the experimenter asked the child to record the keywords again following the same procedure as the baseline speech production. The keywords produced before, during and after the interaction will be compared.

### 3.4. Data analysis

#### 3.4.1. Data extraction and normalization

The vowel portions of the five keywords produced in each recording by the child and the robot were segmented manually by a trained phonetician using Praat (Boersma and Weenink, 2018). The first formant (F1) and second formant (F2) values were extracted at the midpoint of each vowel portion. We adapted the praat script from Stanley and Lipani (2019) to extract the vowel formants automatically.

In order to investigate the adjustment of *f0* parameters in more details, we segmented the child’s production into multiple inter-pause units (IPU). IPU is defined following Levitan and Hirschberg (2011) as a chunk of utterances with pauses in certain duration from one single speaker in one turn, with the adaptation that we adjusted the pause duration from 50 ms to 180 ms, based on previous studies showing that the articulation rate of children (as in our study) in spontaneous speech is significantly slower than adults (as in Jacewicz et al., 2010; Levitan and Hirschberg, 2011). This number was derived empirically from the maximum length of Voice Onset Time of all the recordings. Mean *f0*, maximum *f0,* and minimum *f0* were extracted in each IPU by Praat (Boersma and Weenink, 2018). The *f0* range was calculated as the distance between the minimum *f0* and maximum *f0* in each IPU. We applied the log z-score normalization as in Zhu (2005) to *f0* values.

#### 3.4.2. Statistical analyses

The measurements of phonetic entrainment were to evaluate the similarity of acoustic cues between interlocutors. Regarding the three target parameters (i.e., vowel formant, log mean *f0*, log *f0* range), we compared the differences between the robot and each child across baseline, early production (the first two tasks), late production (the last two tasks), and post-task production. Since the robot’s production was controlled to be consistent throughout the experiment, the differences across time would be contributed by the child.

We first calculated the distance in each parameter (i.e., F1, F2, log mean *f0*, log *f0* range) between the child’s production and the robot’s production. The absolute values of the robot’s production were subtracted from the corresponding values of the child’s production, yielding CRDiff (CRDiff = children’s baseline/early production/late production/post-task production—robot’s production). Linear mixed effects models were then fitted using the “lmerTest” package (Kuznetsova et al., 2017) in R (R Core Team, 2016) to determine whether CRDiffs in vowel formant, log mean *f0*, and log *f0* range were significantly affected by group (autistic vs. TD children) and time period (base vs. early vs. late vs. post). The “effectsize” package (Ben-Shachar et al., 2020) was used to report the standardized coefficient (*β′*) and confidential intervals of the optimal models.

## 4. Results

### 4.1. Vowel formant entrainment

To investigate whether vowel formant adjustment was influenced by subject group and time period, first, a linear mixed effects model was fitted with the CRDiff value as the response variable, the time period and group as fixed effects, and subject and keyword as random effects. The fixed effects and their interaction terms were tested using likelihood ratio tests by adding each variable one at a time for a comparison until the optimal model was chosen.

**Figure 3:**
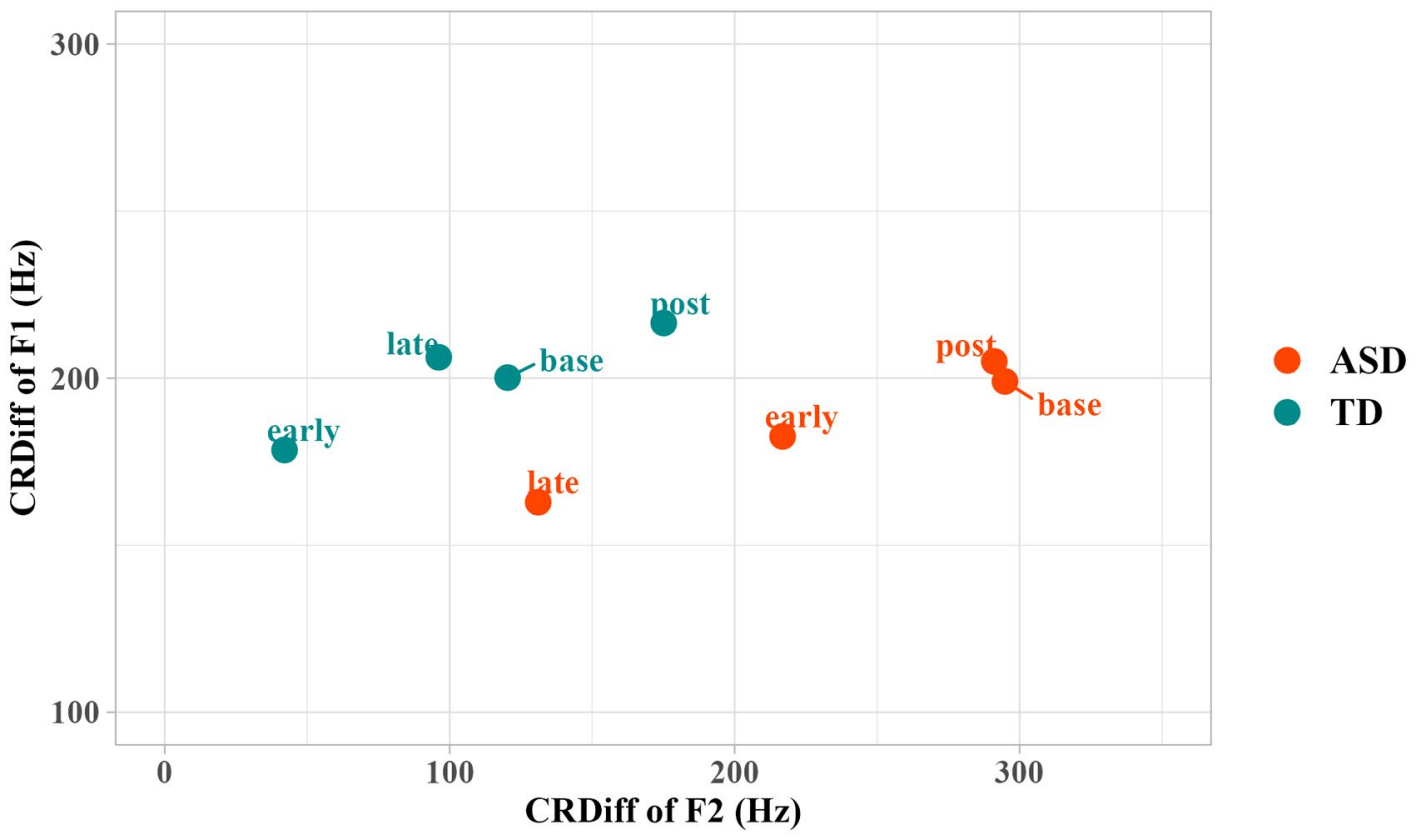
Difference of F1 and F2 between the children and the robot in different time periods. CRDiff = Children’s Production—Robot’s Production.

Regarding RCDiff in F1, only (Time) Period showed a significant effect (Df = 3, *p* = 0.01**). Neither adding Group nor the two-way interaction of Period and Group significantly improved the model. According to Figure 3, both groups of children reduced RCDiff of F1 in the early period. Marginally significant differences were found in comparison between the early and baseline periods (*t* = −1.68, *p* = 0.09; *β′* = −0.06, 95%, CI [−0.13, 0.01]). Autistic children further reduced the RCDiff in the late production, but TD children did not, as indicated by an increase of RCDiff in late period. No significant difference was registered between post-task production and baseline, suggesting that the entrainment only occurred during the interaction.

The adjustment of RCDiff in F2 was more evident. Statistical modeling showed that, by adding Period as a fixed effect, the model significantly improved (*p* < 0.001***). By adding Group and Group * Period interaction, the model improved with marginal significance (*p* = 0.07). Early (*t* = −3.74; *p* < 0.001***; *β′* = −0.22, 95% CI [−0.34, −0.11]) and late (*t* = −5.02; *p* < 0.001***; *β′* = −0.30, 95% CI [−0.42, −0.18]) production showed significant reduction of RCDiff of F2 compared to the baseline, suggesting that both groups of children significantly converged toward the vowel formant of the robot during interaction in terms of F2. We performed *post-hoc* tests using the “emmeans package” (Lenth et al., 2018) to further interpret the Group * Period interaction, and used the estimated marginal means difference (EMMdiff) measures to report the effect size by the “eff_size” function of this package. The *post-hoc* analysis showed a significant reduction in RCDiff of F2 in both early (*t* = 3.74, *p* < 0.01**; Δ = 0.24, 95% CI [0.10, 0.39]) and late (*t* = 5.02, *p* < 0.001***; Δ = 0.33, 95% CI [0.18, 0.48]) periods in the autistic group as compared to the baseline. The TD group showed a trend of reduction in RCDiff of F2, but the reduction did not reach significance. In addition, significant increases of CRDiff (i.e., indicating increasing divergence from the robot) in the post-production relative to early periods (*t* = −3.65, *p* < 0.01**; Δ = −0.24, 95% CI [−0.38, −0.09]) as well as in the post-production relative to late periods (*t* = −4.95, *p* < 0.001***; Δ = −0.32, 95% CI [−0.47, −0.18]) were registered for the autistic group, while TD group showed a significant increase of CRDiff in the post-production relative to early periods (*t* = −3.07, *p* = 0.04*; Δ = −0.22, 95% CI [−0.38, −0.06]), suggesting that the convergence toward the robot occurred specifically during the interaction with the robot interlocutor rather than an adjustment as a result of time/practice with this speech production activity. These results indicate that F2 entrainment occurred more prominently during the early period and started to reduce in the late period for TD children. In contrast, F2 entrainment occurred more prominently in late period for autistic children. Similar to F1, no significant difference between the post-production and the baseline was registered for CRDiff of F2.

In summary, these autistic children entrained in a more gradual way. The degree of entrainment was larger in the late production than the early production in the autistic group. As for TD children, they entrained more prominently in early period and less prominently in late period. The entrainment did not persist in post-task production for either group.

### 4.2. Prosody entrainment

#### 4.2.1. Mean *f0*

**Figure 4:**
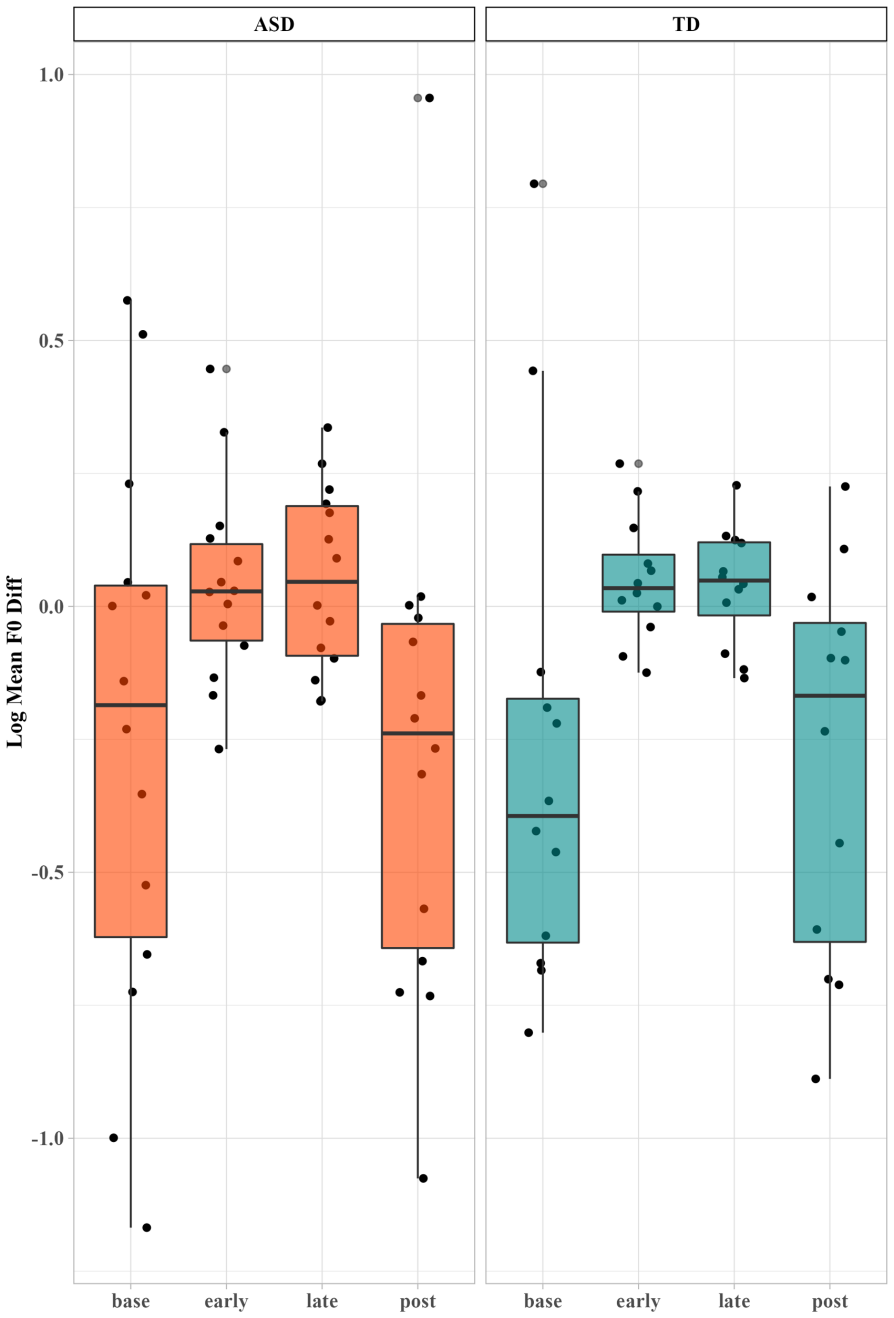
Log mean *f0* difference between the children and the robot across different time periods. Each dot represents a child’s production.

A linear mixed effects model was fitted to test the fixed effect of Period (i.e., early and late), Group and their interaction on CRDiff of log mean *f0* with subject as a random effect. We performed the same modeling procedure as used to analyze vowel formant. Only the fixed effect of Period reached significance (*p* < 0.001***). As we can see from Figure 4, both groups of children reduced the difference of mean *f0* when interacting with the robot (early: *β′* = 0.30, 95% CI [0.23, 0.38]; late: *β′* = 0.33, 95% CI [0.25, 0.40]), and the differences increased in the post-interaction period (*β′* = −0.01, 95% CI [−0.11, 0.09]). No difference between the early and late periods was found, indicating that they entrained as soon as interacting with the robot and that the entrainment remained throughout the tasks.

#### 4.2.2. *F0* range

**Figure 5:**
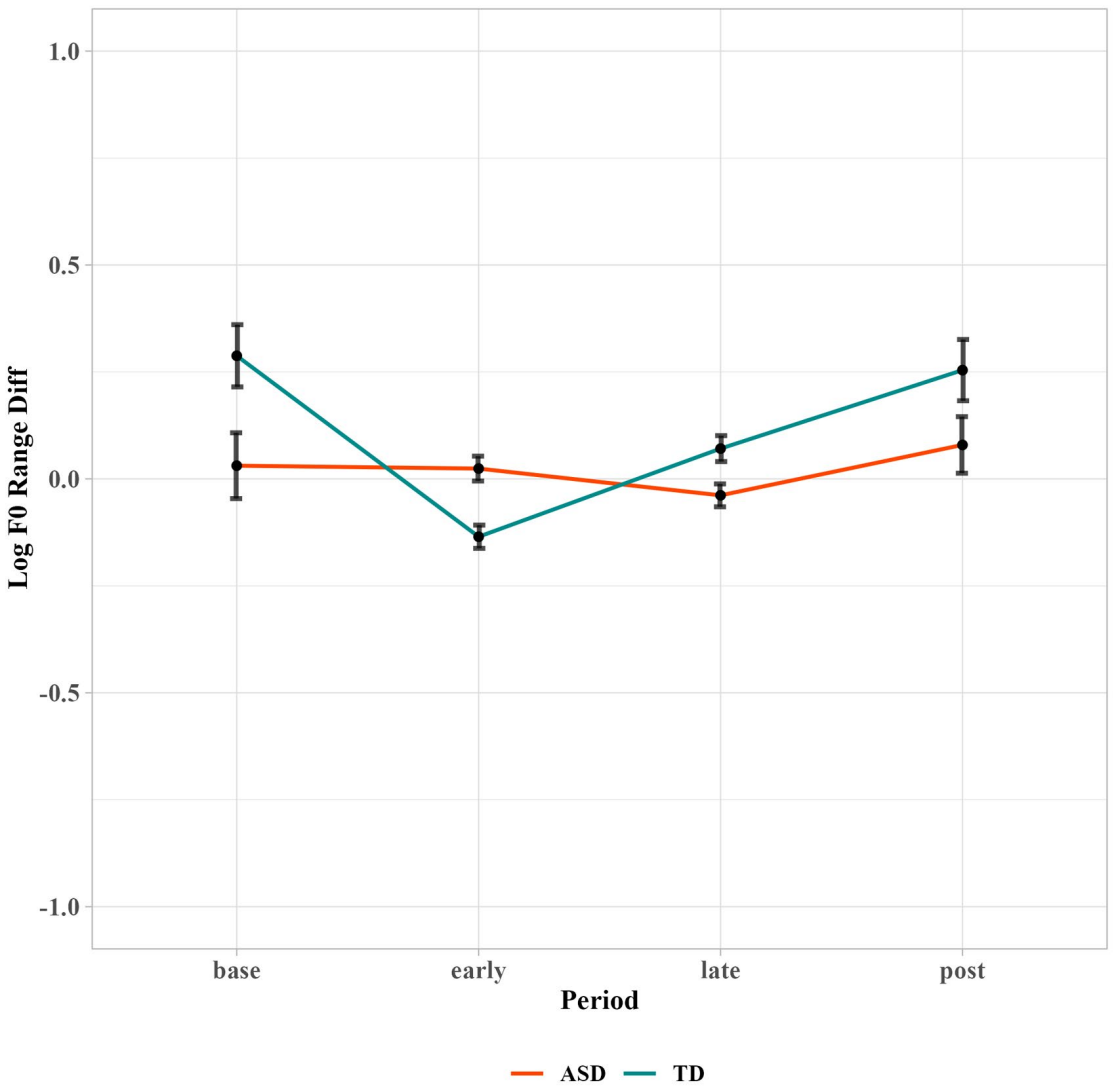
Log *f0* range difference between the children and robot across different time periods.

Regarding the log *f0* range, the linear mixed effects model improved significantly by adding Period (*p* < 0.01**) and the two-way interaction of Period and Group (*p* < 0.01**) as fixed effects. *Post-hoc* analyses to interpret the significant interaction of Period and Group showed that the contribution mainly came from the TD group. The TD group reduced the difference in *f0* range significantly in early period (*t* = 4.9, *p* < 0.001***; Δ = 0.28, 95% CI [0.17, 0.39]) as compared to the baseline. They further adjusted *f0* range difference in late period as compared to early period (*t* = −5.0, *p* < 0.001***; Δ = −0.14, 95% CI [−0.19, −0.08]). By contrast, autistic children did not show much entrainment in terms of *f0* range, as shown in Figure 5. The differences in the *f0* range between the robot and children remained similar when during interactions, suggesting that the participants’ *f0* range were not affected by the interaction. The group difference reached significance in early period (*t* = 3.98, *p* < 0.01*****; Δ = 0.11, 95% CI [0.05, 0.16]), suggesting that at the baseline level, the two groups did not significantly differ in *f0* range difference, but as soon as the TD children started to interact with the robot, their entrainment enlarged the group difference.

As we can see from Figure 5, autistic children showed a similar *f0* range with the robot throughout the time periods, even at baseline prior to interacting with the robot. In order to further our understanding of their *f0* range entrainment, we calculated the standard deviation over each subject’s mean *f0* range in each period, as shown in Table 2. Autistic children showed larger standard deviation in baseline, early, and late periods than TD children. We also noticed a slight fluctuation of f0 range difference from early period to late period for autistic children. We then calculated the number of autistic children showing a reduction of mean *f0* range difference from early to late periods (i.e., more entrainment in late period than early period). Five out of fourteen autistic children exhibited a reduction of differences in late periods while seven out of twelve TD children showed a reduction. This indicated that there were indeed a few autistic children showing phonetic entrainment of *f0* range during interaction. The large individual variation suggested that the reasons behind their lack of *f0* range entrainment were complicated. It is challenging for some autistic children to entrain *f0* range, but not others.

**Table 2:** Standard deviation of *f0* range difference between children and robot in each time period.

Group	Baseline	Early interaction	Late interaction	Post-interaction
ASD	45.97	25.76	28.10	22.15
TD	24.50	22.82	19.32	24.68

#### 4.2.3. Summary

To summarize, both autistic and TD children exhibited a reduction of the mean *f0* differences between them and the robot during their interaction. Regarding *f0* range, our results showed that TD children exhibited reduction of the *f0* range differences from the robot when interacting with the robot, while autistic children showed more individual differences in the phonetic entrainment of *f0* range and did not exhibit adjustment of *f0* range differences from the robot as a group.

## 5. Discussion

We present the first empirical study using a social robot as an interlocutor to investigate whether and how children with and without ASD showed phonetic entrainment in conversations. Since having a social robot interlocutor with speech features and social traits controlled may facilitate phonetic entrainment and its detection in autistic individuals, we expect autistic children may show phonetic entrainment in a more controlled phonetic and social environment, but they may still be different from TD children.

Our study aimed to conduct a more comprehensive investigation examining phonetic entrainment both in vowels and prosody. Specifically, we examined vowel formants and fundamental frequency in the speech production of a group of autistic children and compared these measurements with their TD peers to identify whether or not they would show TD-like phonetic entrainment behaviors. Consistent with our predictions, though autistic children showed some phonetic entrainment, they still exhibited some deficits. Autistic children showed comparable vowel formant entrainment as TD children. Both groups entrained more on F2 than F1. Regarding prosody entrainment, autistic children also showed comparable mean *f0* entrainment as their TD peers. However, while their TD peers showed *f0* range entrainment, the group of autistic children did not exhibit significant convergence toward the interlocutor in terms of *f0* range adjustment, suggesting that entrainment of *f0* range was more challenging and vulnerable for these autistic children even in a more controlled situation.

The fact that autistic children produced vowels in a more extreme way has been documented. In the baseline and post-interaction production, autistic children did produce vowels with larger F2 values, consistent with the results reported by Mohanta and Mittal (2022). These previous findings were interpreted by the authors as attributable to the atypical oral and pharyngeal constriction in autistic individuals when they produced vowels. Nevertheless, our study demonstrated that this atypical mechanism of vowel production did not affect entrainment of vowel formants with an interlocutor producing more controlled speech. Our findings provide support for the claim by Kissine and Geelhand (2019) that autistic population might attend more to the precision of pronunciation. The observed extreme vowel production of autistic population might be due to their overact of articulatory gesture to approach a more precise pronunciation. In our study, the robot produced standard pronunciation of English vowels in a consistent manner, which might be preferred by autistic children and thus triggered their entrainment.

In addition to the findings of vowel formants, autistic children entrained their mean *f0* comparably to their TD peers. This result was inconsistent with some previous studies, where neither autistic nor TD children showed prosody entrainment (Hogstrom et al., 2018; Wynn et al., 2018). As most studies computed the differences between conversation partners to indicate phonetic entrainment, it is very likely that their findings about entrainment or lack of entrainment was actually driven by the adjustment of their interlocutors. In addition, the exaggerated production of the autistic population might trigger atypical judgment of their interlocutors, leading to these interlocutors’ adjustment being more unpredictable. They might entrain to compensate for the larger difference between themselves and the autistic individuals, or they might manipulate their phonetic features away from autistic population because of their atypical production. In our study, the interlocutor (i.e., the social robot) did not adjust its phonetic features no matter whom the robot was talking to. Any manipulation of differences between the dyads came from the child. The consistency of the robot interlocutor made the entrainment more detectable. Another possible reason for this discrepancy in our findings and previous findings could be that social robots were more attractive to children. Previous studies have shown that phonetic entrainment is not merely an automatic imitation process but is mediated by social factors (Coles-Harris, 2017). According to Communication Accommodation Theory (CAT; Giles and Ogay, 2007), positive perception of a conversation partner would reduce the social distance between the individual and the interlocutor and motivate an individual to show entrainment. The attractiveness of a social robot might have reduced its social distance with children and motivated them to entrain phonetically. Yet, an anonymous reviewer pointed out another possibility that the entrainment of prosody might be motivated by a desire for being better understood by the robot. Previous studies have shown that autistic children could differentiate a human voice and a robotic voice (Stanton et al., 2008). But the manipulation of mean *f0* from the robotic voice did not significantly affect children’s performance in a learning task (Molenaar et al., 2021). It is possible that participants entrained to the robot to make themselves understood better. Future studies using both a more human-like voice with natural prosody and a robotic voice without natural prosody may help differentiate the underlying reasons for entrainment in speech prosody. If participants entrain to both a robotic voice without natural prosody, it is likely that they are attempting to build a relationship as lack of natural prosody will not lead to better understandability.

In fact, studies have reported that autistic children showed more interest in interacting with social robots than human beings (Dautenhahn and Werry, 2004; Barakova et al., 2015). Autistic individuals have also been shown to have less interest in human speech voice (Yu and Wang, 2021) and be less able to orient their attention to the human sounds than their TD peers (Čeponienė et al., 2003). It is likely that social robot speaks with a controlled and consistent voice, which may aid their perception and facilitate their phonetic entrainment. On the other hand, autistic individuals have been found to experience multiple difficulties in processing social information such as emotion evaluation (Embregts and Van Nieuwenhuijzen, 2009) and voice identification (Lerner et al., 2013). Previous studies have reported that social robots were usually treated as a human-like category (Eyssel and Kuchenbrandt, 2012; Cohn et al., 2020) and that people tended to compare them with human-beings and evaluate them in a social way—for example, evaluating the ‘membership’ of a robot from the cues of its gender and age (Eyssel and Kuchenbrandt, 2012). In spite of this, the social features of robots are far simpler than humans. Their social complexities demonstrated in interaction, such as facial expressions, social responses, are more limited and controllable. The relatively consistent social information in social robots can reduce the processing load for autistic children. The controlled voice and consistent social information together could have contributed to a more tractable and structured conversational environment, making it more predictable for autistic individuals and easier for them to demonstrate phonetic entrainment.

Apart from documenting comparable phonetic entrainment between the two groups in vowel formants and mean *f0* in this phonetically and socially controlled communication environment, the current study also documented that these autistic children did not show significant *f0* range entrainment as what the TD children exhibited, even with a partner of more controlled speech and social traits. Recall that we also reported larger individual variations of *f0* range differences in the autistic group relative to TD group. It can be inferred that their entrainment behaviors in terms of *f0* range may show more variation compared to the TD group. But as a group, their *f0* range entrainment is not as robust as the TD group. This is in line with findings by Lehnert-LeHouillier et al. (2020) that a few autistic children showing phonetic entrainment, but their statistical results indicated that the autistic children, as a group, did not show comparable entrainment with the TD group. This is also consistent with the emerging literature suggesting a high heterogeneity within the autistic population (Schadenberg et al., 2020). Future research is needed to examine factors that may predict why some autistic individuals are better than the others in phonetic entrainment.

One thing that needs to be noted is that our study examined second language entrainment. Our findings are in line with previous findings of neurotypical L2 speakers, who tended to entrain toward the more prestigious variety when interacting with native speakers (Gnevsheva et al., 2021). In our study, the social robot spoke standard American English, which was more likely to trigger more robust phonetic entrainment from our children participants who spoke English as second language. A few studies have reported signs of relatively intact L2 in autistic populations (Práinsson, 2012; Agostini and Best, 2015), but no study examined phonetic entrainment of their L2 so far. We cannot rule out the possibility that the lack of phonetic entrainment found in previous studies is due to their problems of linguistic entrainment skills in their L1, whereas their L2 entrainment skill might remain relatively intact.

The present study has some limitations, which might need to be addressed in future research. Due to poor speech recognition in Cantonese by the robot, we did not examine phonetic entrainment of the participants’ first language (i.e., Cantonese), which may provide some more direct evidence for phonetic entrainment in human-robot interaction than the evidence in the current study on their L2. It remains to be explored with a Cantonese-speaking social robot in future studies. Moreover, we observed larger individual variation of *f0* range entrainment by autistic children; yet, as the sample size is relatively small, we did not further investigate the contributing factors of their variation. Future work can include more participants and examine the interaction of severity of autism symptoms and phonetic entrainment. In addition, more age groups can be included to obtain a more comprehensive developmental trajectory of children’s phonetic entrainment.

## 6. Conclusion

To conclude, we present the first study investigating phonetic entrainment in autistic children when they interacted with a social robot. The new evidence suggested that these autistic children could entrain phonetically similarly to their TD peers when the interlocutor was controlled in both phonetic and social features. On the other hand, these autistic children did not show entrainment in *f0* range as a group compared to their TD peers, suggesting that phonetic entrainment in *f0* range could be more challenging and vulnerable in autistic children. This study deepens our understanding of autistic children’s conversation behaviors and has implications in designing trainings for autistic children using social robots.

## Data availability statement

The original contributions presented in the study are included in the article/Supplementary material, further inquiries can be directed to the corresponding author.

## Ethics statement

The studies involving human participants were reviewed and approved by Departmental Research Committee of the Hong Kong Polytechnic University. Written informed consent to participate in this study was provided by the participants’ legal guardian/next of kin.

## Author contributions

SC and YH contributed to the conceptualization and experimental design of the study. FZ and TT helped with recruiting participants and data collection. YH ran the statistical analysis under the guidance of SC and wrote the first draft of the manuscript. SC and AC revised the manuscript. All authors contributed to the article and approved the submitted version.

## Funding

This work was supported by Department of Chinese and Bilingual Studies, Faculty of Humanities, the Hong Kong Polytechnic University [UAK7,1-ZVRT; 1-ZE0D; 1-W08C, 1-TA41]. It is also partly supported by the grant from Standing Committee on Language Education and Research (SCOLAR), Education Bureau, HKSAR government [K-ZB2P] and RGC direct allocation grant [A-PB1B].

## Conflict of interest

The authors declare that the research was conducted in the absence of any commercial or financial relationships that could be construed as a potential conflict of interest.

## Publisher’s note

All claims expressed in this article are solely those of the authors and do not necessarily represent those of their affiliated organizations, or those of the publisher, the editors and the reviewers. Any product that may be evaluated in this article, or claim that may be made by its manufacturer, is not guaranteed or endorsed by the publisher.
