# Supplementary material for: Phonetic entrainment in L2 human-robot interaction: an investigation of children with and without autism spectrum disorder
Source: Front Psychol. 2023 Jun 19;14:1128976. doi: 10.3389/fpsyg.2023.1128976 (PMC10315851; doi:10.3389/fpsyg.2023.1128976)
Supplement: Supplementary file 1 [file Table_1.docx]

**Supplementary Material**

Supplementary Figures 1-8: Experimental picture sets in the farm scene. Each pair consists of two pictures. The areas depicting differences were circled and numbered. Figures 1, 3, 5, and 7 were held by the participants, while figures 2, 4, 6, and 8 were held by the robots. Image source: https://doi.org/10.5281/zenodo.3703202. Reproduced under the terms of Creative Commons Attribution 4.0.

Supplementary Figures 9 & 10: Practice picture set in the street scene. Image source: https://doi.org/10.5281/zenodo.3703202. Reproduced under the terms of Creative Commons Attribution 4.0.
