# Supplementary figures and images for: Phonetic entrainment in L2 human-robot interaction: an investigation of children with and without autism spectrum disorder
Source: Front Psychol. 2023 Jun 19;14:1128976. doi: 10.3389/fpsyg.2023.1128976 (PMC10315851; doi:10.3389/fpsyg.2023.1128976)

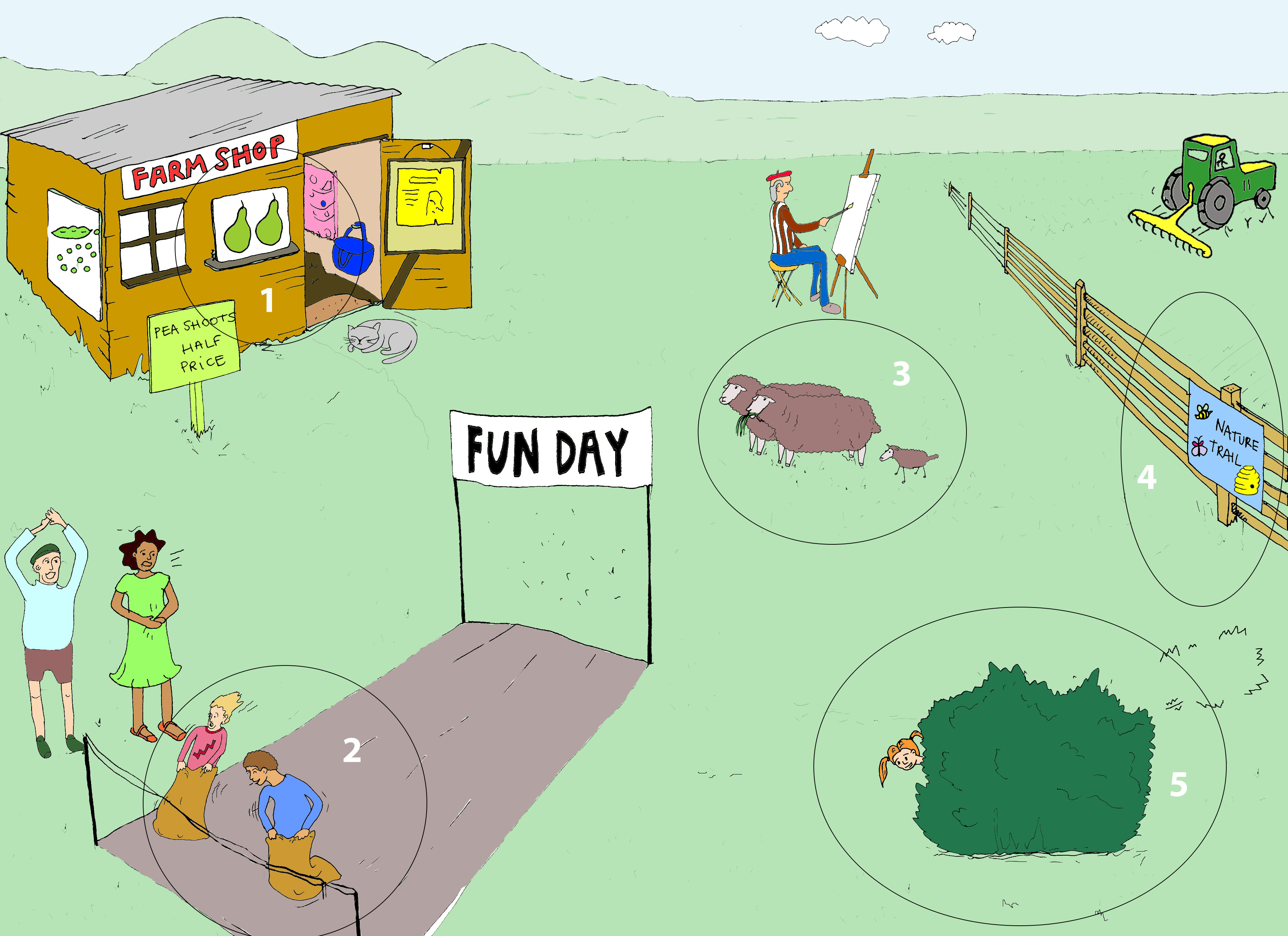

Supplement: Supplementary file 4 [file Image_1.JPEG]

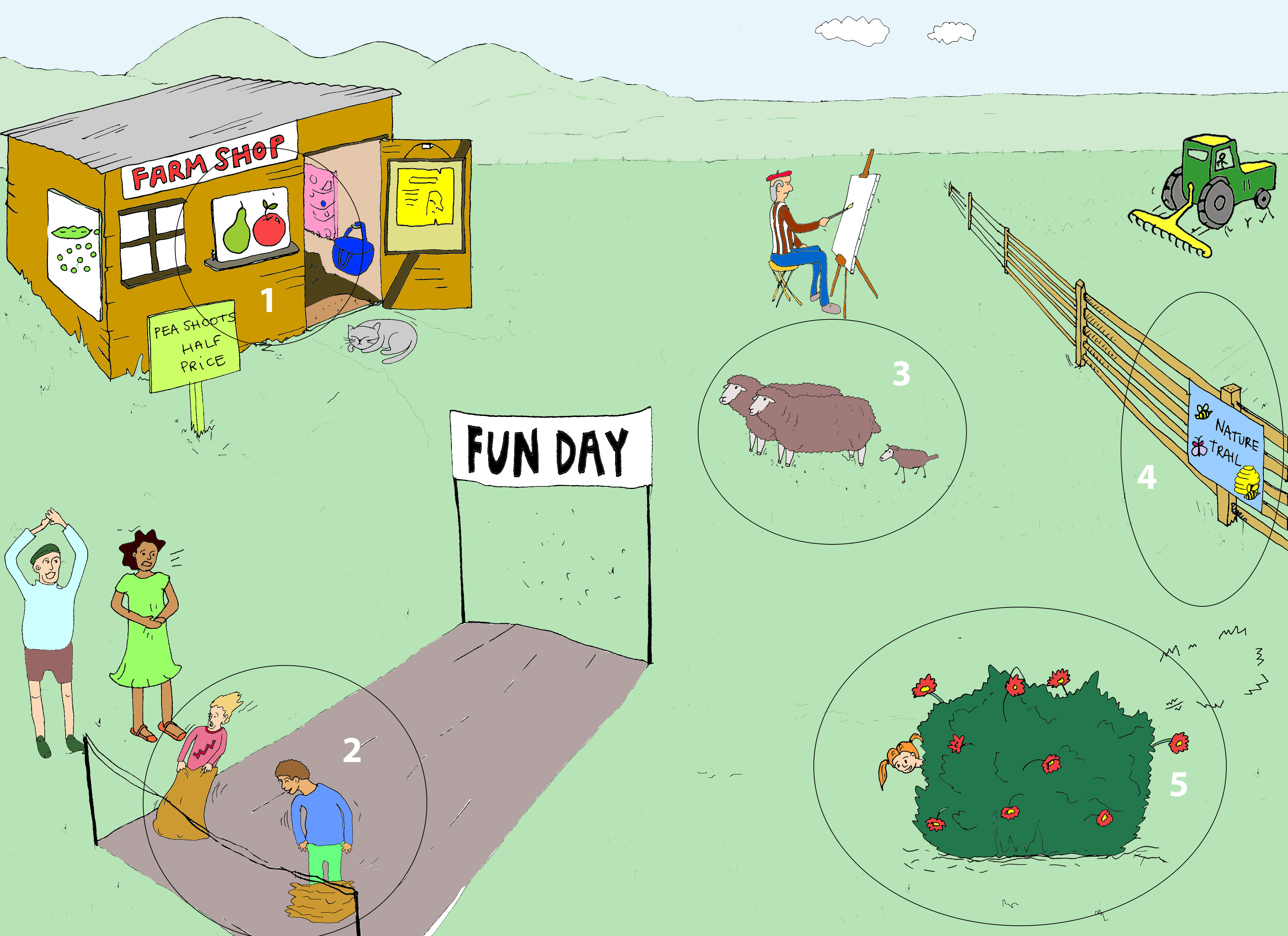

Supplement: Supplementary file 5 [file Image_2.JPEG]

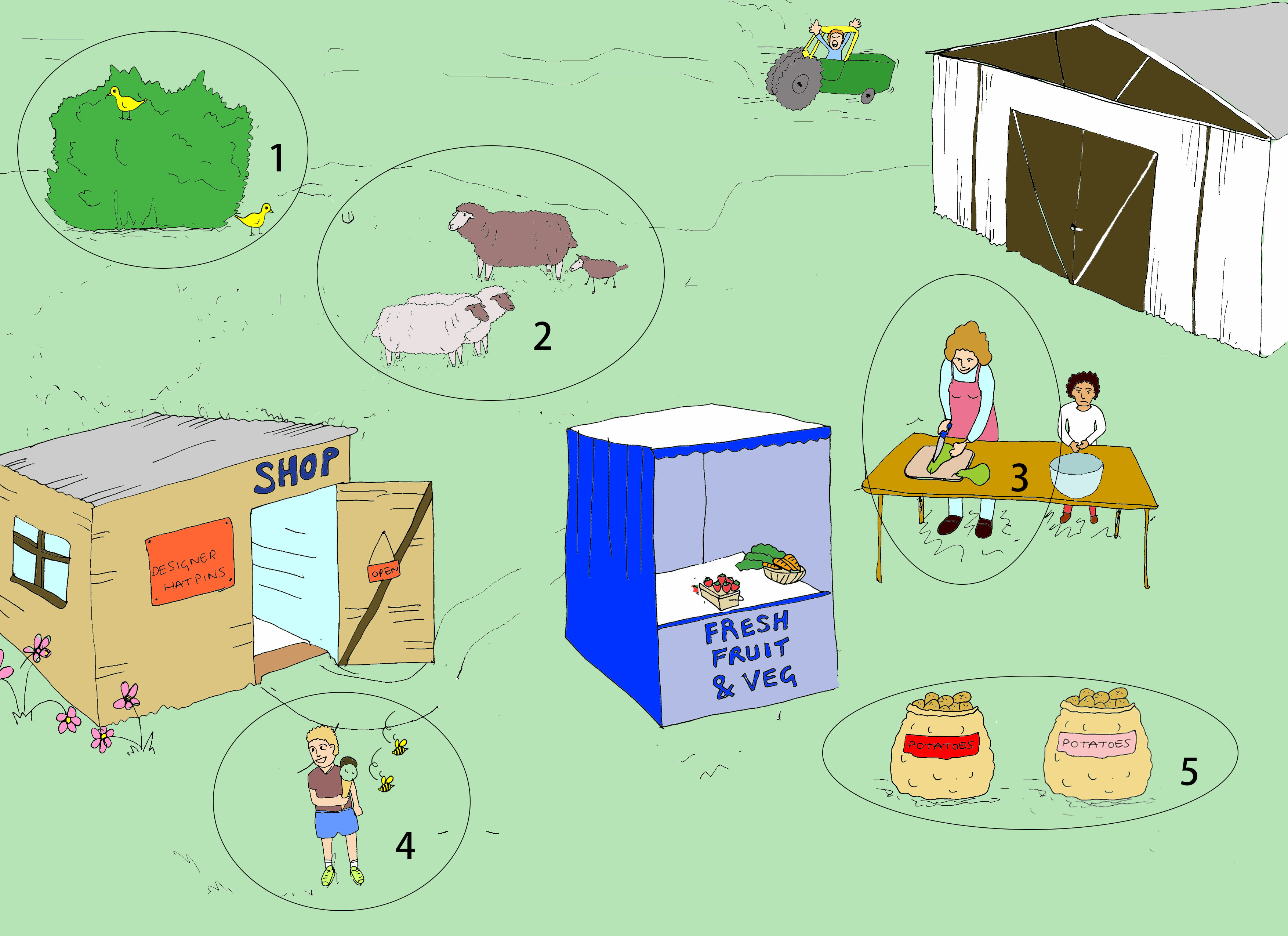

Supplement: Supplementary file 6 [file Image_3.JPEG]

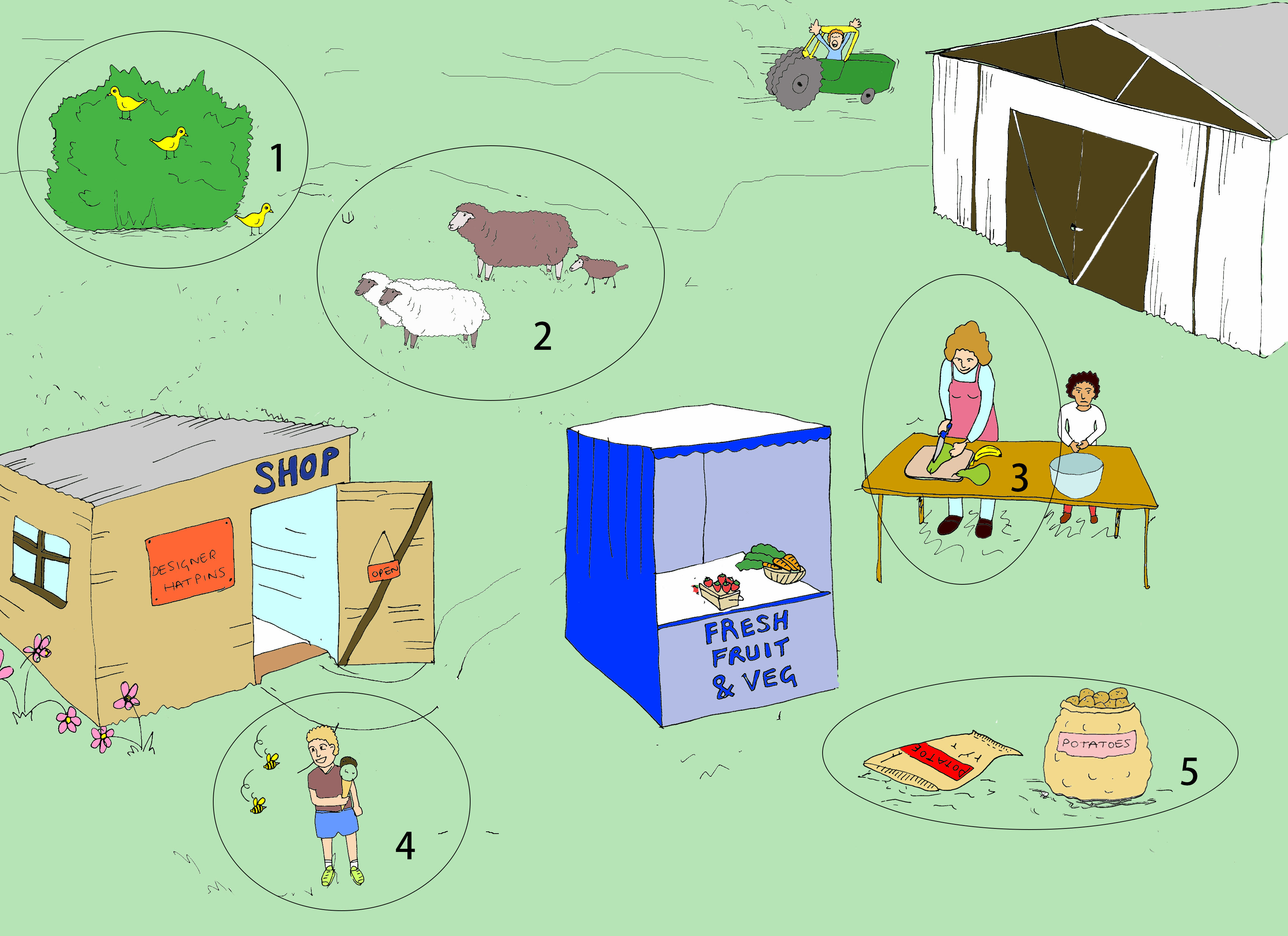

Supplement: Supplementary file 7 [file Image_4.JPEG]

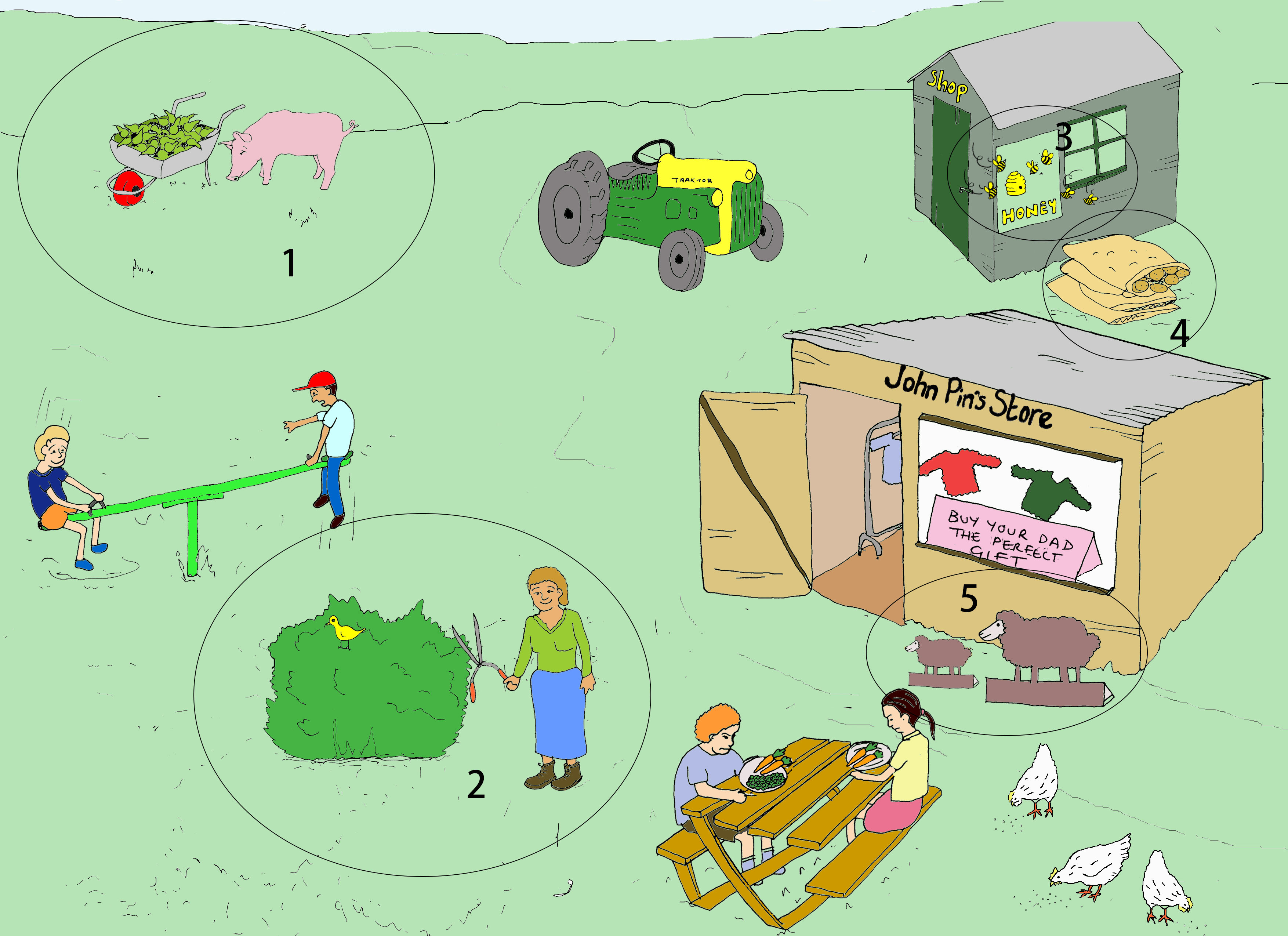

Supplement: Supplementary file 8 [file Image_5.JPEG]

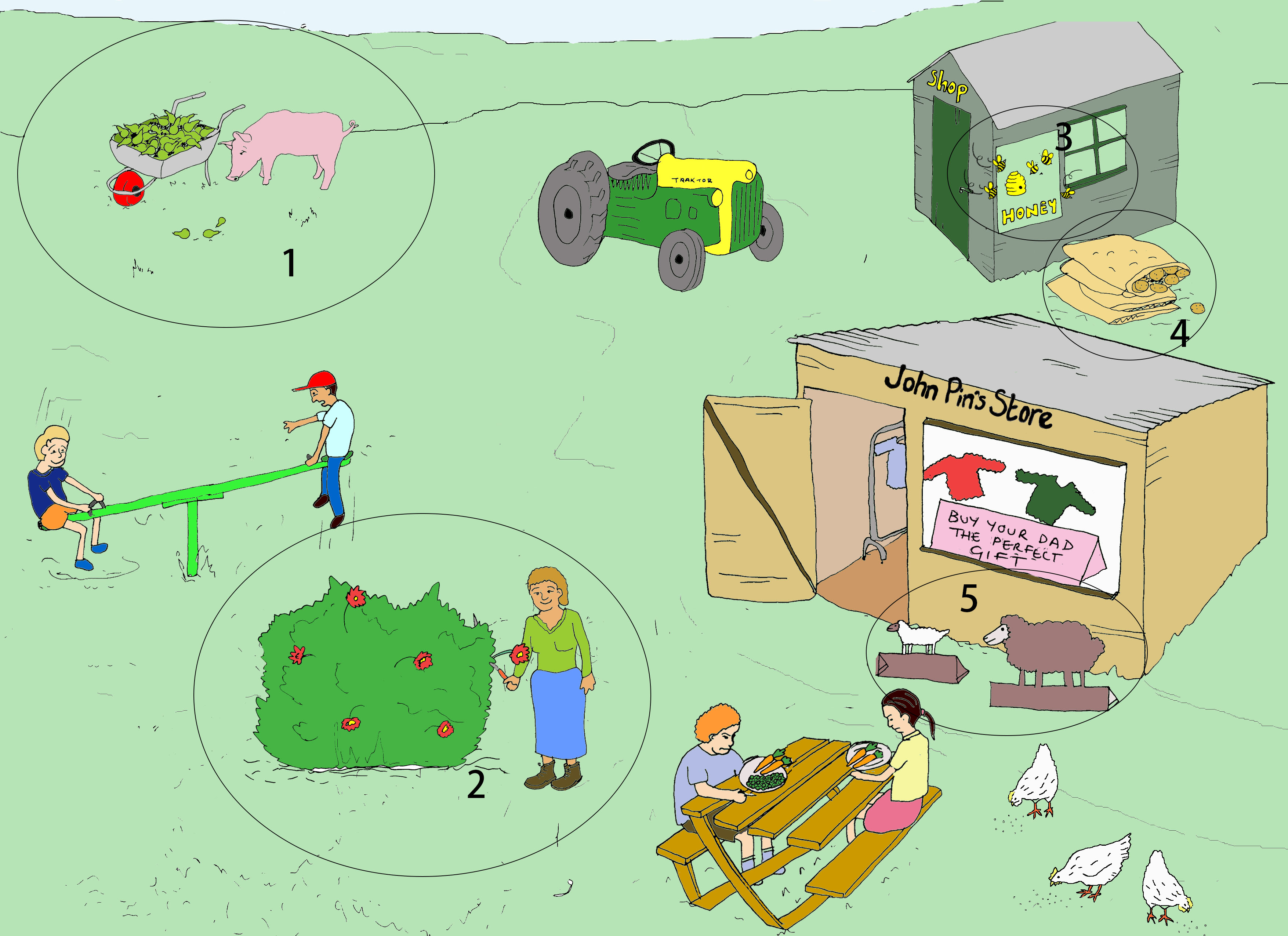

Supplement: Supplementary file 9 [file Image_6.JPEG]

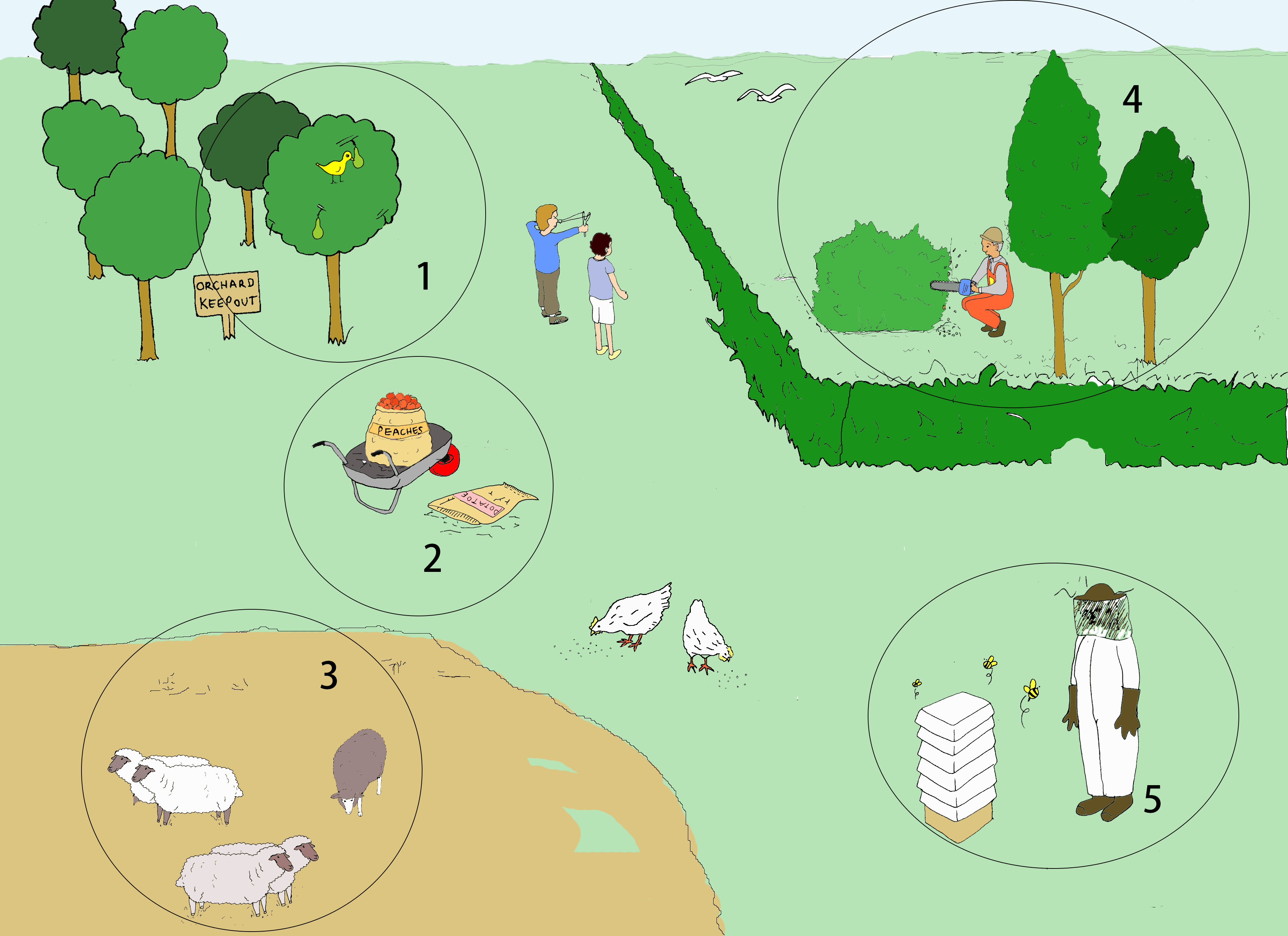

Supplement: Supplementary file 10 [file Image_7.JPEG]

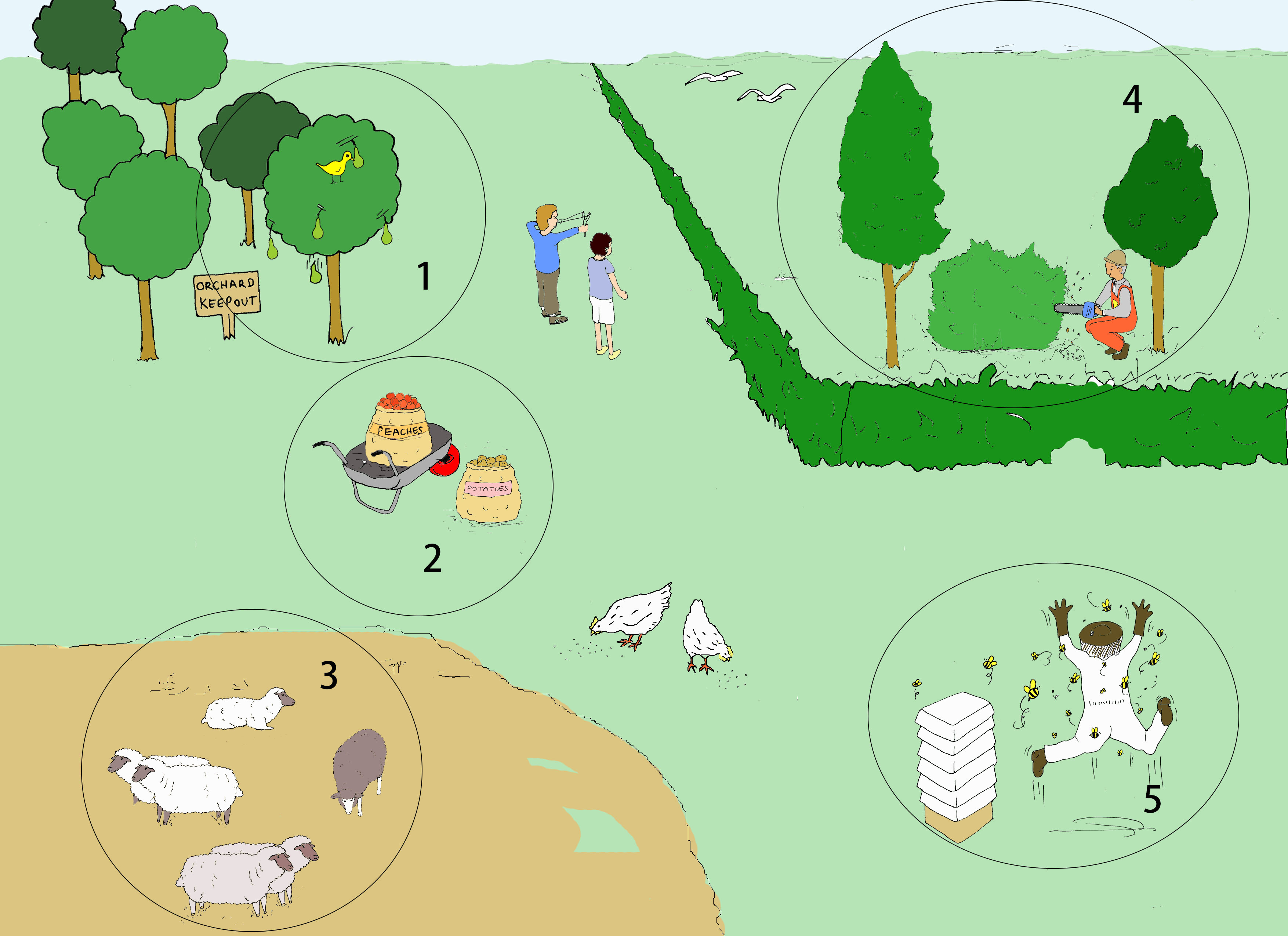

Supplement: Supplementary file 11 [file Image_8.JPEG]

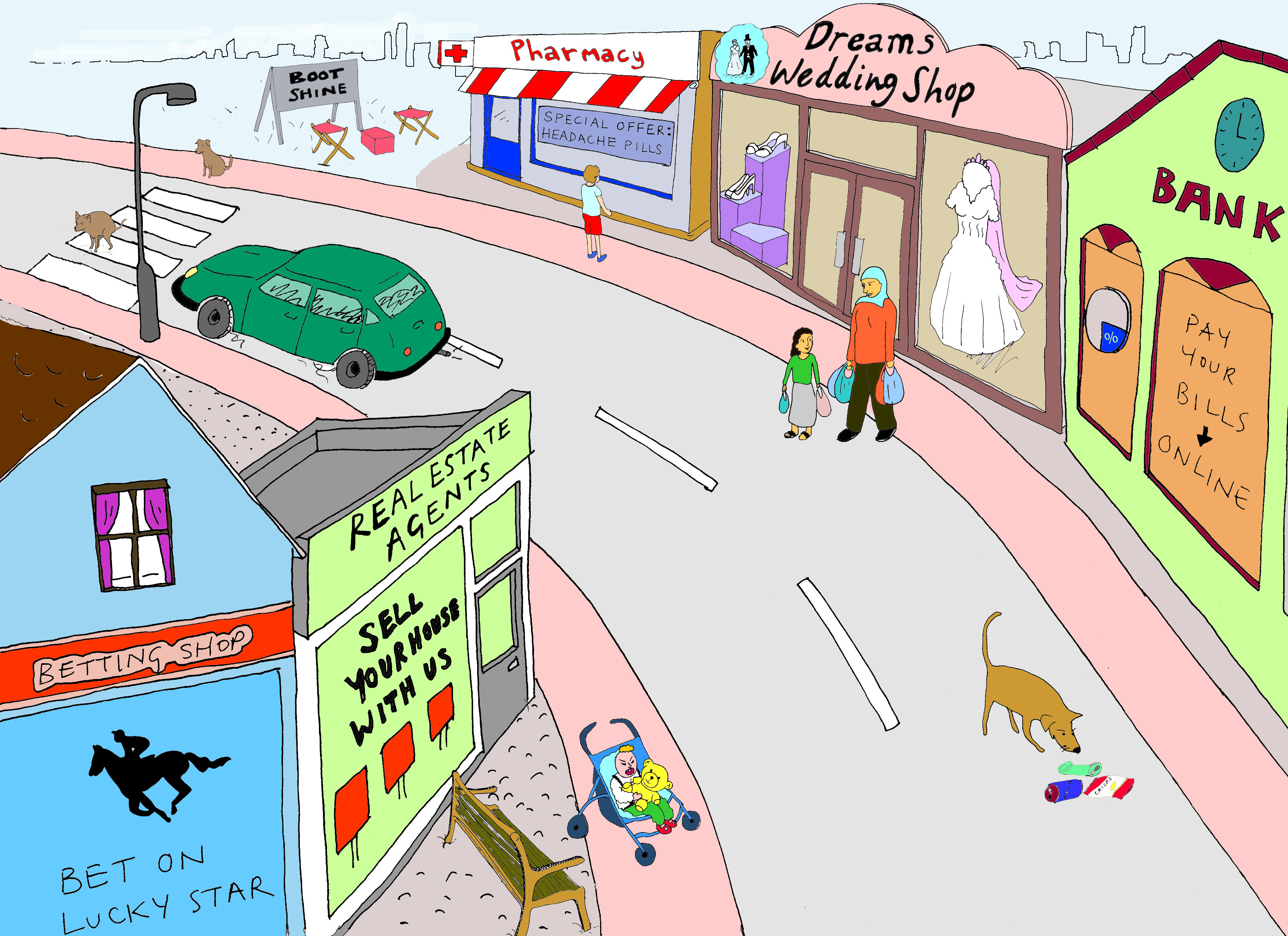

Supplement: Supplementary file 12 [file Image_9.JPEG]

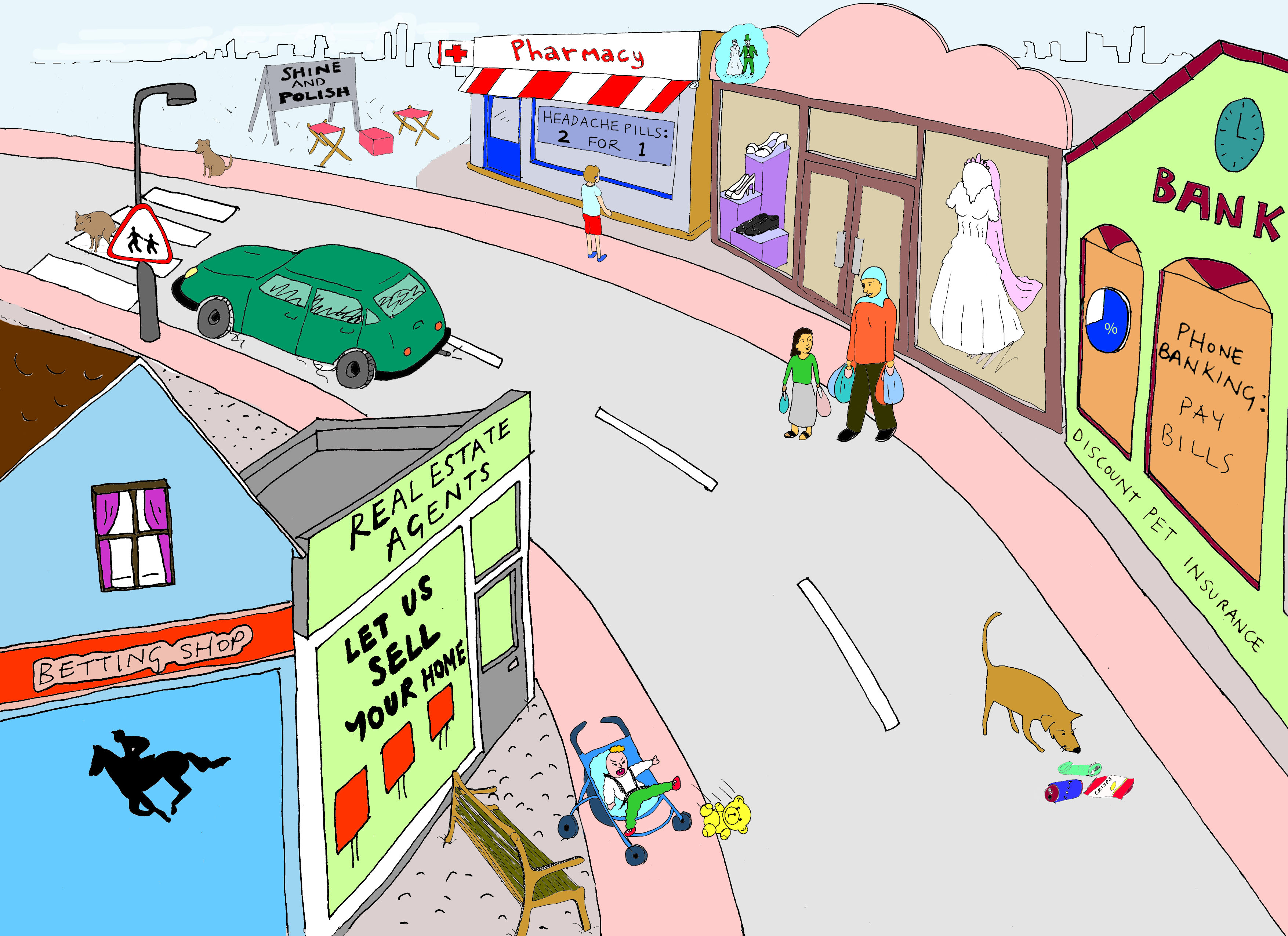

Supplement: Supplementary file 13 [file Image_10.JPEG]
